# Supplementary material for: Multi‐dataset identification of innovative feature genes and molecular mechanisms in keratoconus
Source: J Cell Mol Med. 2024 Sep 19;28(18):e70079. doi: 10.1111/jcmm.70079 (PMC11412914; doi:10.1111/jcmm.70079)
Supplement: Supplementary file 2 — Material S2. [file JCMM-28-e70079-s002.zip › 7.GSEA analysis/6.SNORA74B/KEGG_OOCYTE_MEIOSIS.html]

Details for gene set KEGG\_OOCYTE\_MEIOSIS[GSEA]

|  || Dataset | SNORA74B.group.cls#h\_versus\_l |
| Phenotype | group.cls#h\_versus\_l |
| Upregulated in class | h |
| GeneSet | KEGG\_OOCYTE\_MEIOSIS |
| Enrichment Score (ES) | 0.13576044 |
| Normalized Enrichment Score (NES) | 0.67326206 |
| Nominal p-value | 0.9249012 |
| FDR q-value | 1.0 |
| FWER p-Value | 1.0 |
Table: GSEA Results Summary

  

Fig 1: Enrichment plot: KEGG\_OOCYTE\_MEIOSIS      
 Profile of the Running ES Score & Positions of GeneSet Members on the Rank Ordered List

  

| SYMBOL | TITLE | RANK IN GENE LIST | RANK METRIC SCORE | RUNNING ES | CORE ENRICHMENT || 1 | CAMK2B | na | 141 | 0.835 | 0.0679 | Yes |
| 2 | PPP3R2 | na | 2054 | 0.366 | 0.0442 | Yes |
| 3 | ADCY2 | na | 2866 | 0.309 | 0.0474 | Yes |
| 4 | FBXO43 | na | 2930 | 0.305 | 0.0719 | Yes |
| 5 | CAMK2A | na | 3838 | 0.265 | 0.0686 | Yes |
| 6 | CPEB1 | na | 4805 | 0.232 | 0.0607 | Yes |
| 7 | CHP2 | na | 4857 | 0.230 | 0.0790 | Yes |
| 8 | ADCY5 | na | 6025 | 0.192 | 0.0618 | Yes |
| 9 | ADCY7 | na | 6070 | 0.190 | 0.0769 | Yes |
| 10 | IGF1R | na | 6399 | 0.180 | 0.0830 | Yes |
| 11 | RPS6KA2 | na | 6719 | 0.170 | 0.0884 | Yes |
| 12 | ANAPC1 | na | 6932 | 0.164 | 0.0965 | Yes |
| 13 | CALML6 | na | 7151 | 0.158 | 0.1038 | Yes |
| 14 | CCNE2 | na | 7159 | 0.158 | 0.1172 | Yes |
| 15 | ADCY4 | na | 7191 | 0.157 | 0.1298 | Yes |
| 16 | ESPL1 | na | 7434 | 0.150 | 0.1358 | Yes |
| 17 | CALML5 | na | 8469 | 0.125 | 0.1167 | No |
| 18 | IGF1 | na | 8530 | 0.124 | 0.1256 | No |
| 19 | ITPR2 | na | 8911 | 0.116 | 0.1246 | No |
| 20 | CCNE1 | na | 8992 | 0.114 | 0.1321 | No |
| 21 | AR | na | 10468 | 0.084 | 0.0967 | No |
| 22 | ADCY9 | na | 10911 | 0.076 | 0.0906 | No |
| 23 | ADCY1 | na | 11165 | 0.072 | 0.0895 | No |
| 24 | YWHAE | na | 11170 | 0.072 | 0.0956 | No |
| 25 | SMC1A | na | 11202 | 0.071 | 0.1008 | No |
| 26 | CDK1 | na | 11443 | 0.068 | 0.0998 | No |
| 27 | MAPK12 | na | 11855 | 0.062 | 0.0932 | No |
| 28 | CAMK2G | na | 11900 | 0.062 | 0.0973 | No |
| 29 | PPP2R5A | na | 11922 | 0.061 | 0.1019 | No |
| 30 | PPP2R1B | na | 12000 | 0.060 | 0.1049 | No |
| 31 | STAG3 | na | 12328 | 0.056 | 0.1002 | No |
| 32 | CALM2 | na | 12514 | 0.053 | 0.0995 | No |
| 33 | ADCY6 | na | 12561 | 0.052 | 0.1026 | No |
| 34 | SMC1B | na | 13265 | 0.044 | 0.0861 | No |
| 35 | PGR | na | 13896 | 0.037 | 0.0711 | No |
| 36 | RBX1 | na | 14044 | 0.035 | 0.0699 | No |
| 37 | RPS6KA3 | na | 14434 | 0.031 | 0.0614 | No |
| 38 | YWHAZ | na | 14437 | 0.031 | 0.0640 | No |
| 39 | ANAPC5 | na | 15115 | 0.023 | 0.0464 | No |
| 40 | CDC23 | na | 15160 | 0.023 | 0.0471 | No |
| 41 | ANAPC7 | na | 15214 | 0.022 | 0.0475 | No |
| 42 | CALML3 | na | 15332 | 0.021 | 0.0460 | No |
| 43 | YWHAB | na | 15465 | 0.020 | 0.0439 | No |
| 44 | PTTG2 | na | 15583 | 0.019 | 0.0421 | No |
| 45 | ANAPC4 | na | 15647 | 0.018 | 0.0418 | No |
| 46 | SLK | na | 16082 | 0.014 | 0.0305 | No |
| 47 | PTTG1 | na | 16518 | 0.009 | 0.0187 | No |
| 48 | PPP2R5D | na | 16659 | 0.008 | 0.0154 | No |
| 49 | INS | na | 16698 | 0.007 | 0.0149 | No |
| 50 | ITPR3 | na | 16729 | 0.007 | 0.0147 | No |
| 51 | MAPK3 | na | 16753 | 0.007 | 0.0146 | No |
| 52 | FBXW11 | na | 16931 | 0.005 | 0.0099 | No |
| 53 | MOS | na | 16949 | 0.005 | 0.0099 | No |
| 54 | PPP1CB | na | 17283 | 0.002 | 0.0004 | No |
| 55 | ANAPC13 | na | 17316 | 0.001 | -0.0004 | No |
| 56 | ANAPC11 | na | 17447 | 0.000 | -0.0041 | No |
| 57 | CDC16 | na | 17914 | -0.005 | -0.0172 | No |
| 58 | SKP1 | na | 18228 | -0.009 | -0.0255 | No |
| 59 | PPP1CA | na | 18424 | -0.011 | -0.0302 | No |
| 60 | BTRC | na | 18505 | -0.012 | -0.0314 | No |
| 61 | ADCY8 | na | 18678 | -0.014 | -0.0352 | No |
| 62 | PPP2R5C | na | 19299 | -0.021 | -0.0514 | No |
| 63 | REC8 | na | 19494 | -0.023 | -0.0550 | No |
| 64 | PPP3R1 | na | 19504 | -0.023 | -0.0533 | No |
| 65 | PLCZ1 | na | 19726 | -0.026 | -0.0574 | No |
| 66 | RPS6KA6 | na | 19762 | -0.026 | -0.0562 | No |
| 67 | PPP2R5E | na | 20343 | -0.032 | -0.0702 | No |
| 68 | YWHAG | na | 20368 | -0.032 | -0.0681 | No |
| 69 | CUL1 | na | 20440 | -0.033 | -0.0673 | No |
| 70 | MAPK1 | na | 20606 | -0.035 | -0.0690 | No |
| 71 | PRKX | na | 21076 | -0.041 | -0.0790 | No |
| 72 | PPP2R1A | na | 21264 | -0.043 | -0.0807 | No |
| 73 | YWHAQ | na | 21272 | -0.043 | -0.0772 | No |
| 74 | PPP2R5B | na | 21302 | -0.044 | -0.0743 | No |
| 75 | CHP1 | na | 21507 | -0.046 | -0.0762 | No |
| 76 | PPP1CC | na | 21514 | -0.046 | -0.0724 | No |
| 77 | CDC20 | na | 21595 | -0.047 | -0.0707 | No |
| 78 | MAD2L1 | na | 21747 | -0.049 | -0.0709 | No |
| 79 | CDC27 | na | 21919 | -0.051 | -0.0714 | No |
| 80 | PPP3CB | na | 21942 | -0.051 | -0.0676 | No |
| 81 | CALM1 | na | 22340 | -0.056 | -0.0743 | No |
| 82 | PRKACA | na | 22659 | -0.060 | -0.0783 | No |
| 83 | PPP3CA | na | 23024 | -0.066 | -0.0831 | No |
| 84 | SPDYC | na | 23455 | -0.072 | -0.0893 | No |
| 85 | ANAPC10 | na | 23493 | -0.073 | -0.0841 | No |
| 86 | CALM3 | na | 23658 | -0.075 | -0.0823 | No |
| 87 | ITPR1 | na | 23722 | -0.077 | -0.0776 | No |
| 88 | ANAPC2 | na | 24147 | -0.083 | -0.0827 | No |
| 89 | CCNB2 | na | 24670 | -0.092 | -0.0898 | No |
| 90 | CCNB1 | na | 24727 | -0.093 | -0.0833 | No |
| 91 | PKMYT1 | na | 24796 | -0.095 | -0.0771 | No |
| 92 | CDC26 | na | 24971 | -0.098 | -0.0737 | No |
| 93 | PPP2CA | na | 25151 | -0.101 | -0.0701 | No |
| 94 | SPDYA | na | 25265 | -0.103 | -0.0645 | No |
| 95 | MAD2L2 | na | 25644 | -0.110 | -0.0660 | No |
| 96 | PPP2CB | na | 26235 | -0.122 | -0.0725 | No |
| 97 | SMC3 | na | 26515 | -0.128 | -0.0695 | No |
| 98 | AURKA | na | 26521 | -0.128 | -0.0586 | No |
| 99 | ADCY3 | na | 26777 | -0.134 | -0.0544 | No |
| 100 | CDK2 | na | 27098 | -0.141 | -0.0515 | No |
| 101 | FBXO5 | na | 27730 | -0.158 | -0.0561 | No |
| 102 | RPS6KA1 | na | 27808 | -0.160 | -0.0445 | No |
| 103 | PPP3CC | na | 28713 | -0.186 | -0.0546 | No |
| 104 | BUB1 | na | 29117 | -0.198 | -0.0492 | No |
| 105 | YWHAH | na | 30773 | -0.257 | -0.0748 | No |
| 106 | MAP2K1 | na | 31129 | -0.275 | -0.0614 | No |
| 107 | PRKACB | na | 32349 | -0.344 | -0.0669 | No |
| 108 | CAMK2D | na | 32655 | -0.364 | -0.0443 | No |
| 109 | PRKACG | na | 32698 | -0.368 | -0.0138 | No |
| 110 | CDC25C | na | 33044 | -0.398 | 0.0105 | No |
| 111 | PLK1 | na | 33434 | -0.441 | 0.0374 | No |
Table: GSEA details [plain text format]

  

Fig 2: KEGG\_OOCYTE\_MEIOSIS      
 Blue-Pink O' Gram in the Space of the Analyzed GeneSet

  

Fig 3: KEGG\_OOCYTE\_MEIOSIS: Random ES distribution      
 Gene set null distribution of ES for **KEGG\_OOCYTE\_MEIOSIS**
